# Gene therapy for bladder cancer using E1B-55 kD-deleted adenovirus in combination with adenoviral vector encoding plasminogen kringles 1–5

**DOI:** 10.1038/sj.bjc.6600908

**Published:** 2003-04-29

**Authors:** J-L Hsieh, C-L Wu, M-D Lai, C-H Lee, C-S Tsai, A-L Shiau

**Affiliations:** 1 Institute of Basic Medical Sciences, National Cheng Kung University Medical College, 1 Dashiue Road, Tainan 701, Taiwan; 2 Department of Biochemistry, National Cheng Kung University Medical College, 1 Dashiue Road, Tainan 701, Taiwan; 3 Department of Microbiology and Immunology, National Cheng Kung University Medical College, 1 Dashiue Road, Tainan 701, Taiwan

**Keywords:** E1B-deleted, adenovirus, gene therapy, bladder cancer, p53, plasminogen, kringles 1–5

## Abstract

Mutations or loss of heterozygosity of p53 are detected in approximately 50% of bladder cancers. E1B-55 kD-deleted adenovirus has been shown to kill tumour cells with defective p53 function while sparing normal cells. Here, we examined the cytolytic effect and replication of E1B-55 kD-deleted adenovirus, designated Ad5WS1, on human bladder cancer cell lines with various *p53*status. Ad5WS1 caused more severe cytolytic effect and replicated more efficiently in J82 and TCC-SUP bladder cancer cells carrying mutant *p53*compared with TSGH-8301 and BFTC-905 bladder cancer cells retaining wild-type *p53*. Introduction of dominant negative *p53*into BFTC-905 cells rendered them more susceptible to Ad5WS1-induced cytolysis. Furthermore, cells susceptible to lysis caused by Ad5WS1 were not attributable to their greater infectability by adenovirus. Finally, Ad5WS1 suppressed the growth of TCC-SUP bladder tumour xenografts, which could be augmented when combined with replication-defective adenoviral vector encoding kringles 1–5 of plasminogen (K1–5), an angiogenic inhibitor. Taken together, our results show that E1B-55 kD-deleted adenovirus replicates and hence lyses bladder cancer cells with mutant *p53*much more efficient than those with wild-type *p53*. Thus, E1B-deleted adenovirus may have therapeutic potential, especially in combination with adenoviral vector expressing K1–5, for the treatment of bladder cancer.

Transitional cell carcinoma (TCC) of the bladder is a common cause of death of genitourinary tumours. The generally poor prognosis of advanced bladder cancer indicates the need for new therapeutic modalities. Mutations in the *p53* gene are the most common genetic defect in human bladder cancer, which occur in approximately 50% of bladder cancer and are associated with high-stage, high-grade TCC (Sidransky *et al*, 1991; Esrig *et al*, 1994). In accordance with the important role of p53 in bladder carcinogenesis, loss of heterozygosity of 17p, where *p53* gene is located, is detected in more than 60% of patients with poorly differentiated tumours (Olumi *et al*, 1990). There is a significant correlation between the mutations in the *p53* gene and increased microvessel counts in patients with invasive bladder cancer (Bochner *et al*, 1997). Furthermore, p53 exerts its tumour suppressor effects, in part, through the control of bladder tumour angiogenesis (Grossfeld *et al*, 1997). Therefore, angiogenesis may be one of the therapeutic targets for treating bladder cancer.

The E1B-55 kD protein of adenovirus is able to bind and inactivate p53, and thus allows adenovirus to replicate in cells without the restrain of p53 growth control. Bischoff *et al* (1996) first reported that E1B-55 kD-deleted adenovirus selectively replicated in and lysed cancer cells lacking functional p53 while sparing normal cells. However, subsequent investigations have suggested that replication of this mutant adenovirus may not be entirely dependent upon *p53* status (Rothmann *et al*, 1998; Harada and Berk, 1999). Despite controversy concerning the mechanisms underlying the replication of E1B-55 kD-deleted adenovirus in tumour cells, it is currently employed for cancer therapy, and clinical trials have been promising, especially combined with chemotherapy or radiotherapy (Khuri *et al*, 2000; Habib *et al*, 2001). Notably, no virus was detected in the normal tissue examined in clinical trials (Khuri *et al*, 2000).

Targeting angiogenesis to kill tumour cells is one of the promising therapeutic approaches for cancer. Angiostatin comprising the first four kringles of plasminogen is an endogenous inhibitor of angiogenesis that inhibits the growth of primary and metastatic tumours (O'Reilly *et al*, 1994). A plasminogen fragment containing kringles 1–5 (K1–5) has been shown to exhibit higher antiangiogenic activity and hence higher antitumour effect compared with angiostatin (Cao *et al*, 1999). Targeting tumour vasculature with antiangiogenic gene therapy has become a promising new approach for cancer therapy.

Based on the fact that most bladder cancers have mutation in the *p53*gene, in this study we examined the cytolytic effect of E1B-55 kD-deleted adenovirus on bladder cancer cells with various *p53* status. We demonstrate that this mutant adenovirus exerted a p53-dependent cytolytic effect on bladder cancer cells and its antitumour effect could be augmented when combined with E1-deleted adenoviral vector encoding K1–5. Therefore, our results suggest that E1B-55 kD-deleted adenovirus may have therapeutic potential, especially in combination with replication-defective adenoviral vector expressing K1–5, for the treatment of patients with bladder cancer.

## MATERIALS AND METHODS

### Cell lines and mice

TSGH-8301 (Yeh *et al*, 1988) and BFTC-905 (Tzeng *et al*, 1996) cells carry wild-type *p53*, while J82 and TCC-SUP cells harbour mutant *p53* (Rieger *et al*, 1995; Markl and Jones, 1998). BFTC-173-12 cells were derived from BFTC-905 cells by transfection with V173L, an expression plasmid of a dominant-negative mutant *p53* with a substitution of leucine for valine at codon 173 (Chen *et al*, 1993), followed by selection of stable transfectants with G418 (Chang and Lai, 2001). All the bladder cancer and 293 cells were cultured in Dulbecco's modified Eagle's medium containing 10% fetal bovine serum, 2 mM glutamine and 50 *μ*g ml^−1^ gentamicin. Female BALB/cByJ SmnPrkdc SCID mice at the age of 6–10 weeks used for TCC-SUP tumour cell implantation were obtained from the Laboratory Animal Center of the National Cheng Kung University. The animals were maintained in specific pathogen-free animal care facility under isothermal conditions with regular photoperiods. The experimental protocol adhered to the rules of the Animal Protection Act of Taiwan and was approved by the Laboratory Animal Care and Use Committee of the National Cheng Kung University, in full accordance with the UK Guidelines for the Welfare of Animals in Experimental Neoplasia (Workman *et al*, 1998).

### Construction of recombinant adenoviruses

The E1B-55 kD-deleted adenovirus, designated Ad5WS1, was constructed as described previously (Hsieh *et al*, 2003). The E1-deleted adenoviral vector encoding K1–5, designated Ad/K1–5, was constructed as follows. The coding region of murine K1–5 was generated from pRR1/plasminogen (ATCC, Manassas, VA, USA) by PCR with sense primer 5′-CCC AAG CTT GGA TCC CAT GAA GAG AGT GTA TCT GTC AGA, which introduced a *Hind*III site and an ATG start codon, and antisense primer 5′-GCT CTA GAA TTC TCA TCT GGG GTT TGT TGT ATA GC, which introduced an *Xba*I site and a stop codon. The resulting 1.4-kb DNA fragment was digested with *Hind*III and *Xba*I, and cloned into the adenovirus shuttle vector pAd5L at the *Hind*III/*Xba*I sites, resulting in pAd5L/K1–5. The pAd5L/K1–5 plasmid was cotransfected with pJM17 into 293 cells to generate Ad/K1–5 (Hitt *et al*, 1998). The E1-deleted adenoviral vector Ad/LacZ encoding *β*-galactosidase (*β*-gal) was also used. All the recombinant adenoviruses were propagated and subsequently quantified by plaque assay in 293 cells as previously described (Hitt *et al*, 1998).

### Assays of cytopathic effect (CPE), cell viability and viral replication

Cells cultured in six-well plates were infected with Ad5WS1 at 0.001, 0.01, 0.1 and 1 multiplicities of infection (MOIs) for 2 h. Cells were observed daily for CPE. Once the CPE was evident, which took 5–9 days, cells were stained with 10% formalin/0.05% crystal violet solution. The viable cell numbers were also determined in cells seeded in six-well plates after 3 days of Ad5WS1 infection at an MOI of 2 by trypan blue exclusion. Viral replication was assayed as previously described (Hsieh *et al*, 2003). Briefly, confluent cells in six-well plates were infected with Ad5WS1 at an MOI of 2. Viruses harvested from both the supernatant and cell lysate at 26 h postinfection were pooled and titered on 293 cells by 50% tissue culture infective dose (TCID_50_) method.

### Determination of infectability of human bladder cancer cell lines by adenovirus

To determine the infectability by adenovirus, bladder cancer cells cultured in 24-well plates were infected with various doses of Ad/LacZ in triplicate and incubated for 48 h, after which the cells were stained for *β*-gal activity (Hashimoto *et al*, 1997). Infectability was expressed as a percentage by dividing the number of *β*-gal-expressing cells by the total number of cells from three separate microscopic fields at a magnification of × 200.

### Chorioallantoic membrane (CAM) assay

The angiogenesis assay was carried out as previously described (Shing *et al*, 1985). Ad/K1–5 or Ad/LacZ at a dose of 2 × 10^7^ plaque-forming units (PFU), or PBS was implanted onto the CAMs of a 6-day-old chick embryo through a window made in the egg shell. The CAMs were photographed and analysed for angiogenesis after incubation for 24 h at 37°C.

### Immunohistochemical and vascular staining

At 48 h after infection with Ad/K1–5 at an MOI of 2, TCC-SUP cells were fixed in 3.7% formaldehyde, treated with cold acetone, and quenched in 3.7% H_2_O_2_ for detection of K1–5 expression by immunohistochemical staining using rabbit antibody against human plasminogen kringles 1–3 (K1–3). To evaluate K1–5 expression in tumours, TCC-SUP cells (10^7^ cells) were inoculated subcutaneously (s.c.) into the flank of SCID mice at day 0. At day 10, mice were injected intratumorally with Ad/K1–5 at a dose of 10^8^ PFU for 5 consecutive days. At day 17, tumours were excised and snap frozen. Cryostate sections (4 *μ*m) were prepared and incubated with rabbit antibody against K1–3 followed by detection with the DAKO LSAB 2 System (DAKO, Carpinteria, CA, USA) according to the manufacturer's instructions. After sequential incubation with biotinylated secondary antibody, horseradish peroxidase-labelled streptavidin, and aminoethyl carbazole (AEC) as substrate chromogen, the slides were counterstained with haematoxylin. For vascular staining, the slides were incubated with polyclonal anti-factor VIII (von Willebrand's factor, DAKO), peroxidase-labelled goat anti-rabbit IgG (H & L chain specific, Calbiochem, Darmstadt, Germany), followed by application of AEC, and counterstaining with haematoxylin. Stained blood vessels were counted in three blindly chosen random fields at × 40 magnification, and the mean and s.d. of the counts were calculated.

### Animal studies

TCC-SUP cells (10^7^) were inoculated s.c. into the flank of SCID mice at day 0. At day 8, visible and palpable nodules developed at all injection sites ranging from 25.08 to 47.71 mm^3^, with mean tumour volume of 31.89±6.45 mm^3^. Groups of seven mice were treated intratumorally with Ad5WS1 (10^8^ PFU in 100 *μ*l of PBS) or Ad/K1–5 (2.5 × 10^8^ PFU in 100 *μ*l of PBS) alone for 5 consecutive days, or with Ad5WS1 (10^8^ PFU in 100 *μ*l of PBS) at days 8, 10 and 12 plus Ad5WS1 (10^8^ PFU in 50 *μ*l of PBS) admixed with Ad/K1–5 (2.5 × 10^8^ PFU in 50 *μ*l of PBS) at days 9 and 11. As the injection volume of adenovirus correlates with intratumoral virus distribution, and hence influences therapeutic efficacy (Heise *et al*, 1999), in this study the volume for each intratumoral injection was fixed to 100 *μ*l. All mice were monitored for tumour growth after tumour inoculation. Palpable tumours were measured once a week in two perpendicular axes with a tissue caliper and the tumour volume was calculated as (length of tumour) × (width of tumour)^2^ × 0.45. The mice were killed by intraperitoneal injection of pentobarbital at day 58, when tumours reached approximately 1.7 cm in mean diameter in PBS-treated group.

### Statistical analysis

Tumour volumes and vessel density were compared using an unpaired, two-tailed Student's *t*-test. Any *P*-values less than or equal to 0.05 were regarded as statistically significant.

## RESULTS

### CPE in bladder cancer cells infected with Ad5WS1

**Figure 1:**
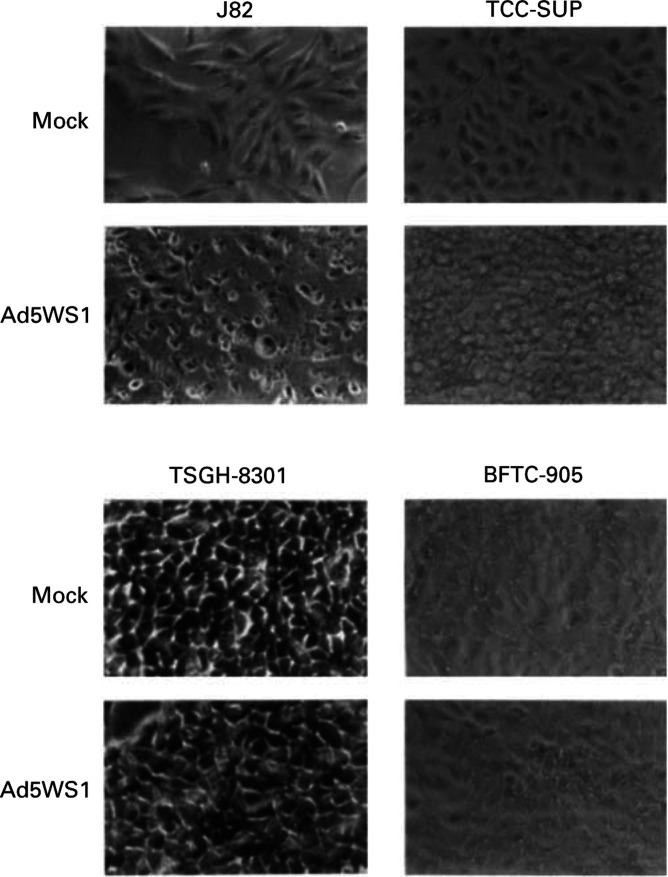
Cytopathic effect (CPE) in human bladder cancer cell lines after Ad5WS1 infection. Cells were infected with Ad5WS1 at an MOI of 2 and monitored for CPE by photomicrographic examination at 5 days postinfection (× 200 magnification).

**Figure 2:**
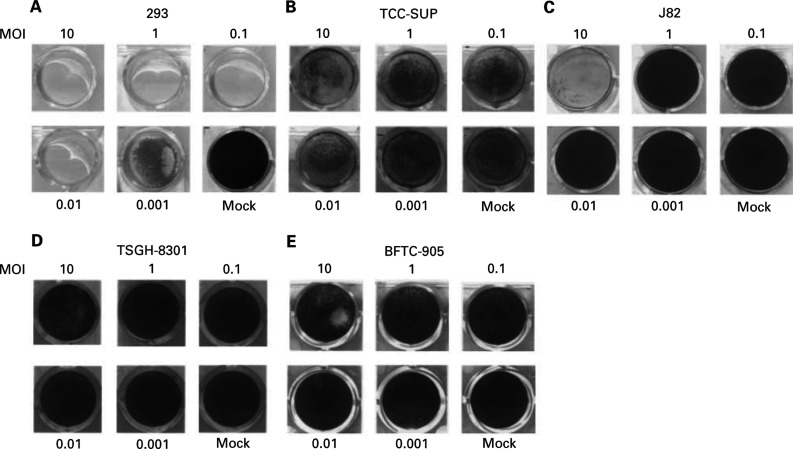
Cytopathic effect (CPE) in human bladder cancer and 293 cell lines after Ad5WS1 infection at different MOIs. Cells were infected with increasing MOIs and monitored for CPE by crystal violet staining at 5–9 days postinfection.

The cytolytic effect of Ad5WS1 on four bladder cancer cell lines with different *p53* status was examined. At an MOI of 2, Ad5WS1 caused CPE in J82 and TCC-SUP cells carrying mutant *p53*, but not in TSGH-8301 and BFTC-905 cells retaining wild-type *p53* at 5 days postinfection (Figure 1

). At 7 days after infection, Ad5WS1 completely lysed 293 cells at MOIs ranging from 0.01 to 10 (Figure 2A

). It lysed TCC-SUP and J82 cells in a dose-dependent manner, with complete cytolysis at an MOI of 10 at 5–7 days postinfection (Figure 2B and C). Nevertheless, even when monitored for 9 days, no significant CPE was observed in TSGH-8301 and BFTC-905 cells infected with Ad5WS1 at the same MOIs (Figure 2D and E). However, at an MOI of 10, Ad5WS1 caused some CPE, albeit only to a very small extent, in TSGH-8301 and BFTC-905 cells (Figure 2D and E). These results indicate that bladder cancer cells carrying mutant *p53* were more susceptible to Ad5WS1-induced cytolysis compared with those carrying wild-type *p53*.

### Replication of Ad5WS1 in bladder cancer cells

**Figure 3:**
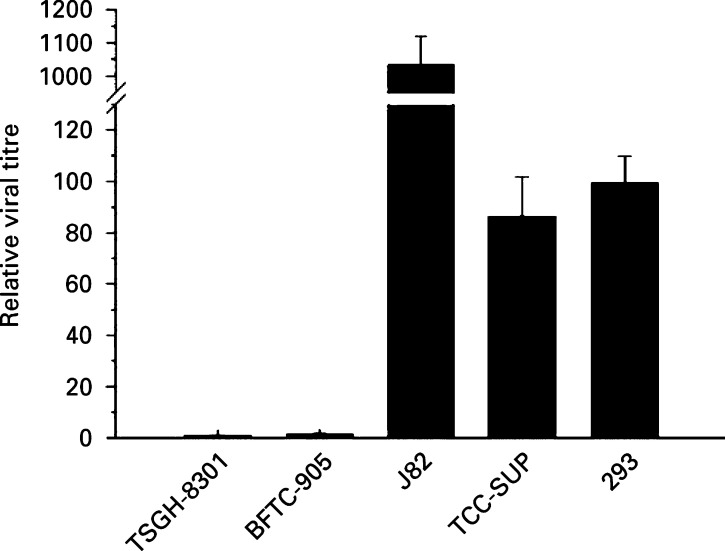
Relative titre of Ad5WS1 on various bladder cancer cell lines compared with 293 cells. Viral replication in bladder cancer and 293 cells infected with Ad5WS1 at an MOI of 2 was determined 26 h later. Viral titres were determined on 293 cells by TCID_50_ method. Results are presented as the titre of the virus determined on 293 cells in each cell line normalised to that in 293 cells. Each value represents the mean of three independent experiments±s.d.

The ability of Ad5WS1 to replicate in four bladder cancer cell lines was assessed at 26 h postinfection. As shown in Figure 3

, Ad5WS1 replicated and produced high titers on both J82 and TCC-SUP cells. Viral yield did not differ between TCC-SUP and 293 cells infected with Ad5WS1. Of note, viral yield produced in J82 cells was found to be 10.34-fold higher over that in 293 cells. However, viral replication was only 0.87% and 1.52% in TSGH-8301 and BFTC-905 cells, respectively, as compared with that in 293 cells. Collectively, Ad5WS1 replicated more efficiently in bladder cancer cells with mutant *p53* than in those with wild-type *p53*.

### CPE in mutant p53 transfectant infected with Ad5WS1

**Figure 4:**
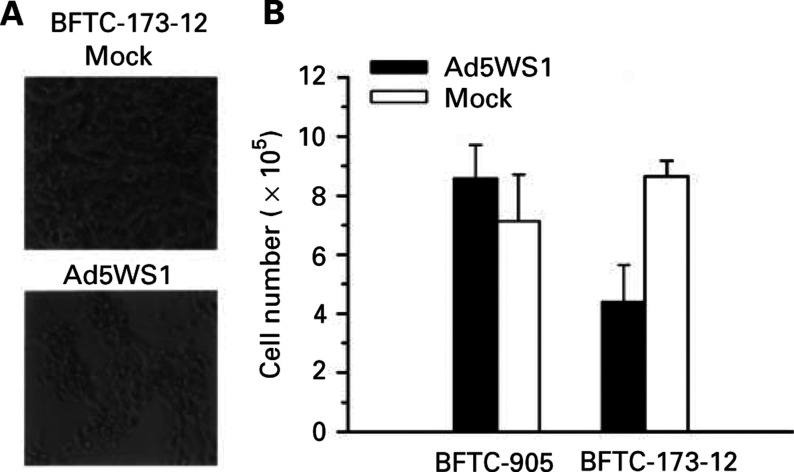
Effects of Ad5WS1 on BFTC-905 cells and BFTC-173-12 cells carrying dominant-negative *p53*. (**A**) CPE in BFTC-173-12 cells infected with Ad5WS1. BFTC-173-12 cells were infected or mock-infected with Ad5WS1 at an MOI of 2 and monitored for CPE by photomicrographic examination at 3 days postinfection (× 200 magnification). (**B**) Cell viability in BFTC-905 and BFTC-173-12 cells after Ad5WS1 infection at an MOI of 2. The viable cell numbers were determined after 3 days by trypan blue exclusion.

Further evidence to support the notion that mutation of *p53* in cells renders them more sensitive to Ad5WS1-induced cytolysis is presented in Figure 4

. BFTC-173-12 cells, which harboured dominant-negative *p53*, exhibited severe CPE following Ad5WS1 infection (Figure 4A), whereas no significant CPE was noticed in parental BFTC-905 cells (Figure 1). In BFTC-173-12 cells infected with Ad5WS1, the number of viable cells decreased to 50% at 3 days postinfection compared with mock-infected cells, whereas the viability of Ad5WS1-infected BFTC-905 cells was not significantly different from mock-infected cells (Figure 4B). In terms of virus replication, viral yields in BFTC-173-12 and BFTC-905 cells were 1.58 × 10^6^ and 1.58 × 10^3^ TCID_50_ ml^−1^ at 26 h postinfection, respectively. These results demonstrate that the mutant p53 transfectant was more permissive for Ad5WS1 replication than parental cells with wild-type *p53*, thus resulting in more cytolysis.

### Infectability of bladder cancer cells by adenovirus

**Figure 5:**
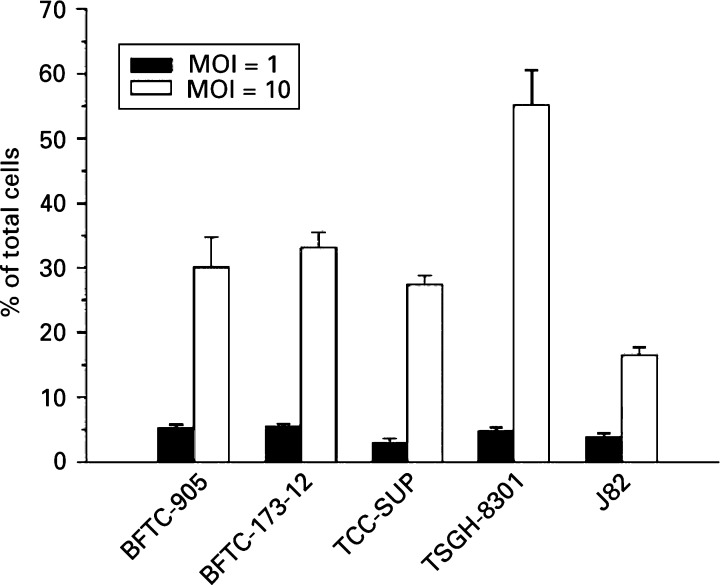
Infectability of human bladder cancer cell lines by Ad/LacZ adenoviral vector. Cells were infected with AdLacZ at MOIs of 1 or 10 in triplicate and incubated for 48 h. Cells were then stained for *β*-gal expression. Infectability was expressed as a percentage by dividing the number of *β*-gal-expressing cells by the total number of cells from three separate microscopic fields at a magnification of × 200. Each value represents mean±s.d. of three determinations. Data were consistent in two separate experiments.

The observed differences in the susceptibility of various human bladder cancer cells to Ad5WS1-induced cytolysis could be attributable to various levels of cell infectability by adenovirus among different cell lines. To address this question, we examined the infectability of the cells by Ad/LacZ, a recombinant adenoviral vector carrying the *β*-gal reporter gene. As shown in Figure 5

, *β*-gal expression was detectable, albeit at different levels, in all of the cells examined after exposure to Ad/LacZ. Furthermore, the infectability of the cells increased when higher doses of Ad/LacZ were applied. The most notable difference was found in TSGH-8301 cells that exhibited three times more infectable with adenovirus compared with J82 cells, which were the least infectable cells. The infectability between BFTC-173-12 and BFTC-905 did not differ, suggesting that introduction of dominant-negative *p53* in the BFTC-905 cells, to obtain the stable BFTC-173-12 transfectant, did not change the infectability of the cells compared with the parental cells.

### Functional characterisation of K1–5 expressed by Ad/K1–5

**Figure 6:**
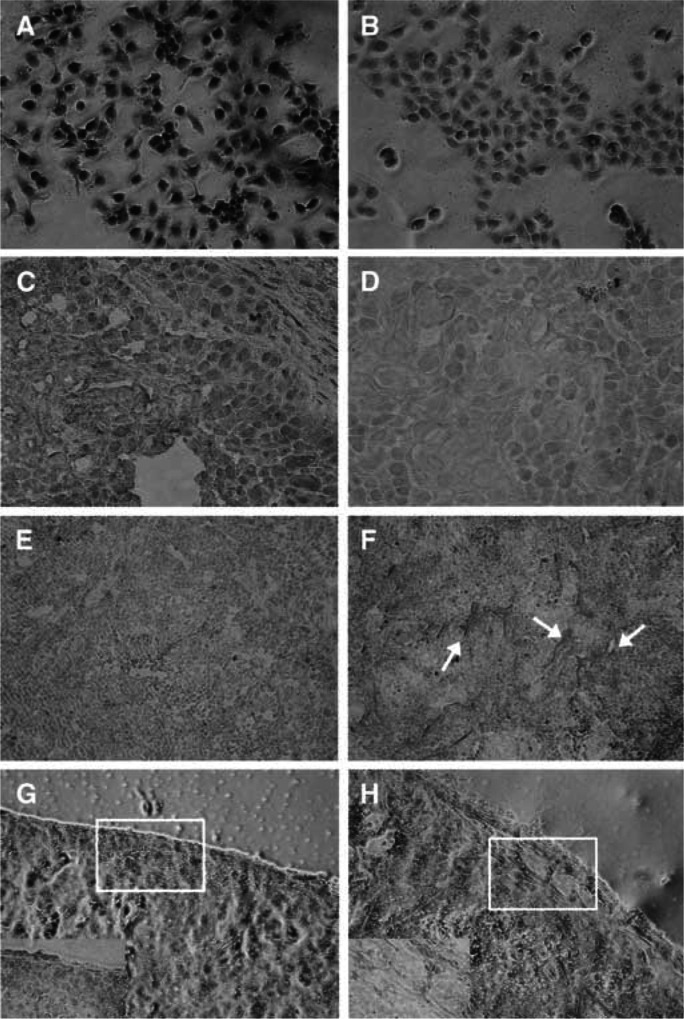
Adenovirus-mediated expression of K1–5 in Ad/K1–5-transduced TCC-SUP cells and tumour xenografts, and its effect on intratumoral vascularisation. Expressions of K1–5 in TCC-SUP cells (**A**) infected with Ad/K1–5 at an MOI of 2 or (**B**) mock-infected were determined by immunostained with rabbit antibody against human K1–3 (× 200 magnification). Expressions of K1–5 in TCC-SUP bladder tumour xenografts after treatment with (**C**) Ad/K1–5 or (**D**) PBS (× 200 magnification). Reduction of intratumoral vascularisation (**E**, **G**) in Ad/K1–5-treated tumours, but not in (**F**, **H**) PBS-treated tumours determined by immunostained with factor VIII (× 100 magnification). The arrows indicate the location of microvessels. The insets in G and H represent the magnified area (× 400 magnification).

**Figure 7:**
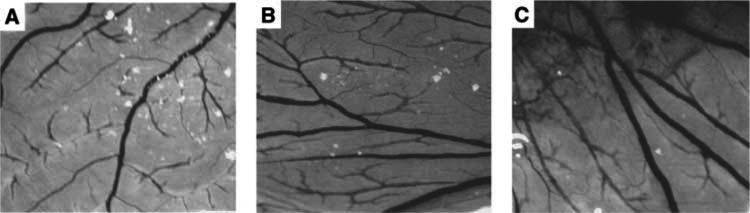
Chorioallantoic membrane (CAM) assay of Ad/K1–5. Chorioallantoic membranes of 6-day chick embryos were treated with (**A**) Ad/K1–5 (2 × 10^7^ PFU), (**B**) Ad/LacZ (2 × 10^7^ PFU) or (**C**) PBS. The CAMs were photographed after incubation for 24 h at 37°C.

Expression of K1–5 was detectable in more than 80% of TCC-SUP cells transduced with Ad/K1–5 (Figure 6A

) compared with untransduced cells (Figure 6B). Expression of K1–5 in tumours was further examined in TCC-SUP tumour-bearing SCID mice treated with Ad/K1–5. By immunohistochemical staining, K1–5 protein was detectable in the tumour xenografts from Ad/K1–5-treated (Figure 6C), but not PBS-treated mice (Figure 6D). Furthermore, tumour xenografts were analysed for microvessel morphology and vascularisation by staining for factor VIII. Tumour sections from Ad/K1–5-treated mice appeared much less vascularised (Figure 6E and G) than their PBS-treated counterparts (Figure 6F and H). The vessel density underneath tumour capsules was markedly reduced in Ad/K1–5-treated (12±2.65 vessels per field) compared with PBS-treated (32.67±2.52 vessels per field) tumours (*P*<0.001). Furthermore, K1–5 encoded by Ad/K1–5 inhibited angiogenesis in the CAM assay. Ad/K1–5 inhibited the growth of embryonic new blood vessels (Figure 7A

) compared with Ad/LacZ-treated (Figure 7B) or PBS-treated (Figure 7C) CAMs. Ad/K1–5 inhibited angiogenesis in four of six CAMs, while only one of six CAMs in Ad/LacZ-treated group revealed suppression of neovascularization, and none was found in PBS-treated CAMs. Taken together, these results indicate that the expressed K1–5 mediated by Ad/K1–5 gene transfer was detectable and displayed antiangiogenic activity *in vitro* and *in vivo*.

### Antitumour effects of Ad5WS1 and Ad/K1–5

**Figure 8:**
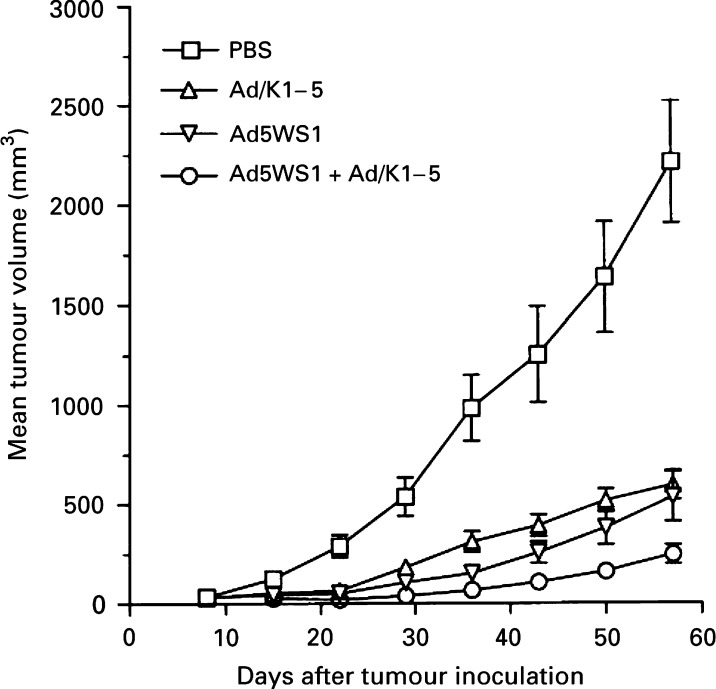
Effects of Ad5WS1 and Ad/K1–5, singly or in combination, on human TCC-SUP bladder tumour xenograft growth. TCC-SUP cells (10^7^) were inoculated s.c. into the flank of SCID mice at day 0. At day 8 when visible and palpable nodules developed at all injection sites, groups of seven mice were treated intratumorally with Ad5WS1 (10^8^ PFU) or Ad/K1–5 (2.5 × 10^8^ PFU) alone for 5 consecutive days, or with Ad5WS1 (10^8^ PFU) for 5 consecutive days plus Ad/K1–5 (2.5 × 10^8^ PFU) at days 9 and 11. Data are presented as mean±s.e.

The antitumour effects of Ad5WS1 alone or combined with Ad/K1–5 were examined in pre-established human TCC-SUP bladder tumour xenografts. As shown in Figure 8

, TCC-SUP tumours from either Ad5WS1-treated (*P*=0.00027) or Ad/K1–5-treated (*P*=0.00024) mice were significantly smaller than those from PBS-treated mice. Furthermore, at day 58 combination treatment of Ad5WS1 and Ad/K1–5 inhibited tumour growth compared with Ad5WS1-treated (*P*=0.05) or Ad/K1–5-treated (*P*=0.0018) mice. Of note, complete tumour regression was observed in one of seven established tumours in the combined treatment group, but not in the remaining three treatment groups (data not shown).

## DISCUSSION

In the work described here, Ad5WS1 caused more severe CPE and produced higher viral yield in cells carrying mutant *p53* compared with those carrying wild-type *p53*. At an MOI equal to or less than 2, the CPE caused by Ad5WS1 in human bladder cancer cell lines appeared to be p53-dependent. However, at very high viral doses and an extended period of infection, Ad5WS1 also caused some CPE, albeit only to a very small extent, in BFTC-905 and TSGH-8301 cells with wild-type *p53*. Studies along this line demonstrated that E1B-deleted adenovirus can infect tumour cells with functional p53 at higher viral doses, while kill those carrying nonfunctional p53 at much lower doses in hepatocellular carcinoma and lung cancer cell lines (Vollmer *et al*, 1999; You *et al*, 2000). It has been demonstrated that when sufficient E1B-55 kD-deleted viral particles are delivered and sufficient target cells are in the S phase of the cell cycle, the virus may replicate regardless of the *p53* status of target cells (Goodrum and Ornelles, 1997). In the viral replication assay shown here, compared with cancer cells harbouring wild-type *p53*, the observed CPE caused by Ad5WS1 in bladder cancer cells carrying mutant *p53* corresponded to as much as 1000-fold difference in virus yield. Our results also indicate that infectious E1B-deleted adenoviruses may still be produced to a small extent in cells harbouring wild-type *p53*, despite the absence of an obvious CPE, which were in accordance with previous findings (Bischoff *et al*, 1996; Ganly *et al*, 2001). Since most related reports do not include quantitative evidence of viral replication and the production of new virus in the cancer cell lines studied, the relation between *p53* status and viral replication or CPE still awaits clarification.

The infectability of cell lines by adenovirus has been reported to be dependent on the presence of coxsackie and adenovirus receptor (CAR) and *αβ* integrins, which are two cell membrane components involved in viral attachment and internalisation (Wickham *et al*, 1993; Bergelson *et al*, 1997). It has been shown that a wide spectrum of CAR levels exists among human bladder cancer cells, which may correlate with their sensitivity to adenovirus infection (Li *et al*, 1999). Our results argue against the possibility that the infectability levels of the human bladder cancer cell lines by adenovirus contribute to their susceptibility to cytolysis induced by Ad5WS1. In this study, Ad5WS1 replicated efficiently and caused cytolysis in TCC-SUP but not in BFTC-905 cells regardless of their similar level of infectability by adenovirus. Furthermore, TSGH-8301 cells were resistant to Ad5WS1-mediated cell death despite their high infectablity by adenovirus. Ad5WS1 replicated most efficiently in J82 cells, which, however, were least infectable with adenovirus among the cell lines examined. A recent study has also shown that CAR expression on TCC-SUP cells is about four times higher than on J82 cells (van der Poel *et al*, 2002). Therefore, our results clearly show that the susceptibility of human bladder cancer cells to Ad5WS1-induced cytolysis was not attributed to differences in their infectability by adenovirus. To confirm the correlation between *p53* status and susceptibility to E1B-55 kD-deleted adenovirus, BFTC-905 cell line and its derivative BFTC-173-12 transfectant, in which p53 function has been ablated by expression of a dominant-negative *p53* allele, were used in parallel to determine the susceptibility of the bladder cancer cells to Ad5WS1-induced cytolysis. Our results demonstrate that disruption of p53 in bladder cancer cells rendered them more susceptible to Ad5WS1-induced cytolysis. We also showed that the higher sensitivity to Ad5WS1 replication in BFTC-173-12 cells compared with BFTC-905 cells was not attributable to the increase of infectability, because BFTC-173-12 cells were as infectable by Ad/LacZ as were the parental BFTC-905 cells. To this end, we have recently shown that Ad5WS1-induced cytolysis is dependent on the transcription activity of p53 in hepatocellular carcinoma cells (Hsieh *et al*, 2003). Furthermore, introduction of the hepatitis B virus *X* gene into liver cells sensitises them to cytolysis induced by Ad5WS1 through the disruption of p53 transcription activity.

Although antitumour activity of E1B-55 kD-deleted adenovirus has been demonstrated in a wide range of different human tumours *in vitro* and *in vivo*, the mechanism underlying its oncolytic activity remains controversial. Recently, E1B-55 kD-deleted adenovirus has been shown to exert cytotoxic effect in seven established human bladder cancer cell lines (van der Poel *et al*, 2002) *in vitro*. However, this study did not further investigate the underlying mechanism. Ries *et al* (2000) have shown that loss of p14^ARF^, a negative regulator for the MDM2 expression, facilitates the replication of E1B-55 kD-deleted adenovirus in tumour cells retaining wild-type *p53*. This finding supports the therapeutic use of this mutant adenovirus not only in tumours with mutant *p53* but also in tumours with lesions within the p53 pathway. In bladder cancer, alterations affecting the p53 pathway, including p53, MDM2, p21^WAF1^ and p14^ARF^, are frequent events, which may be important diagnostic and prognostic markers in patients with bladder cancer (Lianes *et al*, 1994; Markl and Jones, 1998). As MDM2 ubiquitinates p53 and targets it for proteosomal degradation, it inactivates the function of p53. In a number of human cancers, the *MDM2* gene is amplified or overexpressed, leading to inadequate levels of p53. Thus, p53 may be indirectly inactivated by mechanisms other than *p53* mutations in some bladder cancers. It is plausible that tumour cells in which high levels of MDM2 suppress p53 may allow E1B-deleted adenovirus replication. BFTC-905 cells have been shown to overexpress MDM2 mRNA (Cheng *et al*, 1995), which may explain in part why Ad5WS1 at high doses caused CPE, albeit to a very small extent, in bladder cancer cells harbouring wild-type *p53*. Further analysis of the p53 pathway in bladder cancer cells may answer part of this question. The molecular basis for the growth differences of E1B-deleted adenovirus within different tumour and normal cell types remains to be determined.

In a number of clinical trials, E1B-55 kD-deleted adenovirus is well-tolerated by numerous routes of administration. However, as a single agent, its therapeutic efficacy for refractory solid tumours has been limited. Combination therapy seems to have greater promise in improving its antitumour activity. In the present study, we employed Ad5WS1 in combination with Ad/K1–5, an E1-deleted, replication-defective adenoviral vector expressing angiogenic inhibitors, for the treatment of TCC-SUP bladder tumour xenografts. Because antiangiogenesis therapy is likely to be most effective in a low tumour burden state (Bergers *et al*, 1999), we started the treatment in mice bearing tumours ranging approximately from 25 to 48 mm^3^. Mice with low tumour burdens have been used in previous reports for evaluation of adenovirus-mediated antiangiogenic gene therapy (Im *et al*, 1999; Sauter *et al*, 2000). We found that combined therapy of Ad5WS1 and Ad/K1–5 was superior to either treatment alone in inhibiting tumour growth. E1B-55 kD-deleted adenovirus has been shown to act as a helper virus and augment the expression of the accompanying cytokine genes delivered by replication-defective adenoviral vector (Motoi *et al*, 2000). Although antiangiogenic therapy targets genetically stable endothelial cells in the tumour vasculature, tumour cells with mutated *p53* are less susceptible to apoptosis under hypoxic conditions, resulting in reducing their reliance on vascular supply, and hence their responsiveness to antiangiogenic therapy (Yu *et al*, 2002). As tumour cell populations may be heterogeneous in terms of genetic mutations in clinical settings, combination therapy targeting to different types of tumour cells would be expected to enhance therapeutic efficacy. To this end, the strategy of combination therapy of oncolytic E1B-deleted adenovirus with adenoviral vector encoding angiogenic inhibitors is more desirable in clinical settings, as cancer cells with nonfunctional p53 would be more susceptible to Ad5WS1-induced cytolysis than those with wild-type *p53*, while tumours retaining functional p53 more responsive to antiangiogenic therapy because of more vascular dependence. Several phase I/II clinical trials of oncolytic adenoviruses for head and neck, pancreatic, ovarian and gastrointestinal carcinomas have been carried out. However, oncolytic adenoviral therapy has not been applied to the patients with bladder cancer. Owing to the fact that intact urothelium can function as an effective barrier, bladder is suitable for intravesical instillation of oncolytic E1B-deleted adenovirus or adenoviral vectors. In line with this notion, intravesical instillation of adenoviral vectors has been shown recently to be safe, feasible and biologically active in patients with bladder cancer (Kuball *et al*, 2002).

In conclusion, our results demonstrate that E1B-55 kD-deleted adenovirus caused more severe cytolytic effect and replicated more efficiently in bladder cancer cells with mutant *p53* than in those with wild-type *p53*. In the bladder xenograft animal model, it also exerted antitumour effects, which could be augmented by combining with replication-defective adenoviral vector expressing K1–5. Therefore, our results suggest that E1B-55 kD-deleted adenovirus may have therapeutic potential, especially in combination with adenoviral vector expressing K1–5, for the treatment of patients with bladder cancer.
